# Reproductive factors and pancreatic cancer risk: a Norwegian cohort study

**DOI:** 10.1038/sj.bjc.6604095

**Published:** 2007-11-13

**Authors:** I Heuch, B K Jacobsen, G Albrektsen, G Kvåle

**Affiliations:** 1 Department of Mathematics, University of Bergen, Johannes Bruns Gate 12, N-5008 Bergen, Norway; 2 Institute of Community Medicine, University of Tromsø, N-9037 Tromsø, Norway; 3 Section for Epidemiology and Medical Statistics, Department of Public Health and Primary Health Care, University of Bergen, PO Box 7804, N-5020 Bergen, Norway; 4 Centre for International Health, University of Bergen, PO Box 7804, N-5020 Bergen, Norway

**Keywords:** pancreatic neoplasms, pregnancy, lactation, reproductive history

## Abstract

A cohort of 63 090 Norwegian women born 1886–1928 was followed more than 38 years, and relations between reproductive factors and risk of pancreatic cancer were explored; 449 cases were recorded at ages 50–89 years. Age at menopause showed a moderately positive association with risk (rate ratio (RR)=1.08 per 2 years delay in menopause; 95% confidence interval (CI)=1.00–1.17). Neither parity nor duration of breastfeeding showed significant associations with risk after adjusting only for demographic factors. With mutual adjustment, however, parity became positively associated (RR=1.13 per delivery; 95% CI=1.05–1.22) while duration of breastfeeding was inversely associated (RR=0.87 per 12 months; 95% CI=0.78–0.97). These associations lessened in magnitude with increasing age, and were essentially absent above age 80 years. Risk was raised among women reporting at least one abortion, but no trend was seen with number of abortions. Together with previous studies, the findings raise questions about the role of chance, but do not exclude hormonal factors related to breastfeeding and pregnancy from affecting pancreatic cancer risk.

Among Norwegian women, only cancers of lung, breast, colon and ovary cause more deaths than pancreatic cancer (Cancer Registry of Norway, 2006). Female sex hormones may be involved in the aetiology of pancreatic cancer (Robles-Diaz and Duarte-Rojo, 2001), with experimental studies indicating that carcinogenesis may be promoted as well as inhibited by steroid hormones. Findings regarding expression of oestrogen receptors have been inconsistent (Satake *et al*, 2006), although both oestrogen and progesterone receptors have been found in normal and neoplastic pancreatic tissue (Robles-Diaz and Duarte-Rojo, 2001).

Lower incidence rates of pancreatic cancer in women than men, particularly at premenopausal ages, have suggested that sex hormones may be protective in women (Bourhis *et al*, 1987). However, although Norwegian women traditionally had much lower incidence than men, this difference has narrowed considerably in the last 20 years (Cancer Registry of Norway, 2006), while the remaining contrast may reflect risk factors such as smoking.

Some studies have indicated overall positive (Miller *et al*, 1980; Ji *et al*, 1996) or inverse relations with parity (Kreiger *et al*, 2001; Skinner *et al*, 2003). Others showed a higher (Karlson *et al*, 1998; Navarro Silvera *et al*, 2005) or lower (Hanley *et al*, 2001; Teras *et al*, 2005) risk among women with many children only or even no association with parity (Duell and Holly, 2005; Prizment *et al*, 2007). Breastfeeding was covered in only two studies (Skinner *et al*, 2003; Lo *et al*, 2007) while menstrual factors have shown inconclusive findings (Prizment *et al*, 2007).

We investigated reproductive factors in relation to pancreatic cancer during a 38-year follow-up of a cohort of Norwegian women born over the period 1886–1928. Results after shorter periods of follow-up were reported previously for parity (Kvåle *et al*, 1994) and duration of breastfeeding (Kvåle and Heuch, 1988).

## MATERIALS AND METHODS

The cohort consisted of women who participated in a breast cancer-screening programme in 1956–1959 in the Norwegian counties of Nord-Trøndelag, Aust-Agder and Vestfold. A detailed questionnaire, completed in personal interviews, covered reproductive factors (Kvåle, 1989). No distinction was made between spontaneous and induced abortions, but in the birth cohorts considered most abortions would have been spontaneous. Where relevant, information on age at menopause was also recorded, defined as age at last menstruation. Among the 85 063 women resident in the three counties aged 32–74 years by 1 January 1961, a total of 63 090 women attended and were interviewed.

Using personal identification numbers, data on reproductive factors were, with approval from the Norwegian Data Inspectorate, linked to follow-up information on deaths and emigration from Statistics Norway and to cancer diagnoses from the Cancer Registry of Norway.

Follow-up extended from 1 January 1961 until 31 December 1998 and terminated at diagnosis of pancreatic cancer, at age 90 years, death or emigration. Only five cases of pancreatic cancer occurred under age 50 years, and in the present study the follow-up started at age 50 years. Of the 62 613 women followed, 33 769 died and 148 emigrated or were removed from the population registry. They contributed a total of 1 505 163 person-years, with a mean follow-up time of 24 years. A total of 449 cases of pancreatic cancer (ICD 7, code 157) were recorded, among them 231 were histologically verified. Among the remaining cases, the diagnosis was based on surgical reports in 99 cases, X-ray examinations in 60 cases, clinical examinations in 31 cases, autopsy in 4 cases and death certificates in 24 cases.

Incidence rates were assessed by log-linear Poisson regression analysis (Preston *et al*, 1996), taking into account the person-years for the various risk factor levels. Age was regarded as a time-dependent variable. Estimated linear relations were based on the original values recorded, in years for age and months for duration of breastfeeding, and not the grouped values used to study categorical effects.

Age at menopause was studied in the 22 145 women known to be postmenopausal at the time of interview, among whom 192 cases of pancreas cancer occurred. Because of missing values, the numbers in analyses of other risk factors varied slightly. Measurements of height and weight were carried out separately in 1963–1975 in a subcohort. In analyses adjusted for body mass index (BMI), defined as weight/(height)^2^, follow-up started 2 years after the measurements to reduce a possible effect of disease on weight; these included 49 055 women, with 329 cases.

Information on individuals' smoking habits, a potential confounder, was not available. Absolute age-specific incidence rates of lung cancer (ICD 7, codes 162 and 163), during follow-up, were used to assess the extent of smoking. To compare birth cohorts, only the age intervals 60–64, 65–69, 70–74 and 75–79 years were considered, with a total of 393 cancer cases.

## RESULTS

Pancreatic cancer showed only minor variation in incidence over birth cohorts after accurate adjustment for age (Table 1) but differed moderately between the three counties. Risk was rather similar in broad occupational categories of the women or of their husbands, regarded as indicators of socioeconomic status (results not shown).

No association was found between age at menarche and risk (Table 2). Age at menopause showed a moderately positive association, particularly for women experiencing menopause above age 50 years (Table 2). Excluding women who reported oophorectomy, hysterectomy or other operations on the ovaries or uterus showed similar risk estimates among the remaining 19 728 women with natural menopause (results not shown). The number of fertile years, defined as the period between menarche and menopause, did not show any additional association with risk above that seen with age at menopause (rate ratio (RR)=1.07 per 2 years in a linear analysis including 187 cases; 95% confidence interval (CI)=0.99–1.14).

No statistically significant relations were found with parity or total duration of breastfeeding, adjusting for demographic variables only (Table 3). With adjustment for breastfeeding, however, a significant positive relation emerged with parity, nulliparous having the lowest risk (RR=0.61, 95% CI=0.42–0.90) compared to all parous women. Women with three or more deliveries had an elevated risk (Table 3). Similarly, a significant inverse relation was found with duration of breastfeeding after adjusting for parity. Parous women who had not breastfed had a higher risk than any category who did, and also within each category of parity. Rate ratios for those who breastfed compared to those who did not were 0.60, 0.56, 0.85, 0.54 and 0.39, for women of parity 1, 2, 3, 4 and ⩾5, respectively. Risk declined further for quite long breastfeeding periods (Table 3). Excluding women with no breastfeeding still indicated an inverse although slightly weaker linear relation with the total breastfeeding period (RR=0.90 per 12 months of breastfeeding; 95% CI=0.80–1.01); no linear interaction could be demonstrated between parity and duration (*P*=0.55). Age at menopause was only weakly associated with parity and breastfeeding in this cohort (Kvåle, 1989) and further adjustment for age at menopause had little effect.

Analyses of age at delivery in uniparous women and for ages at first or last delivery in multiparous women showed no association with risk (Table 4). Women reporting at least one abortion showed a higher risk than those without abortions (Table 5), but no trend was seen with the number of abortions.

Linear analyses were performed for parity, duration of breastfeeding and age at menopause within broad categories defined by attained age and birth cohort. No significant interactions were found with age or birth cohort, but the effects of all three risk factors essentially disappeared above about 80 years (Table 6). Risk estimates for duration of breastfeeding based on linear analyses were remarkably stable over birth cohorts.

Analyses restricted to histologically verified cases produced similar linear estimates with parity (RR=1.12 per delivery; 95% CI=1.01–1.24) and duration of breastfeeding (RR=0.88 per 12 months; 95% CI=0.75–1.02). For age at menopause, a slightly stronger estimate was found (RR=1.11 per 2 years; 95% CI=0.99–1.24). Body mass index showed no association with risk, and adjusting for it did not influence relations with age at menopause, parity or breastfeeding, and nor materially did occupational category.

Absolute incidences per 100 000 person-years of lung cancer for the birth cohort 1886–1900 were 6, 10, 16 and 28 for the age intervals 60–64, 65–69, 70–74 and 75–79 years, respectively. For the birth cohort 1901–1910, the corresponding rates were 10, 15, 20 and 39; and for the birth cohort 1911–1928, 38, 65, 72 and 90.

## DISCUSSION

In this cohort study, the risk of pancreatic cancer was influenced by age at menopause and, after mutual adjustment, by parity and duration of breastfeeding. The study design, with a complete and lengthy follow-up, essentially eliminated selection bias. Data on risk factors were collected before follow-up, and incomplete information on reproductive variables, possibly affecting women born after 1910, should mostly attenuate risks. Women with early menopause were overrepresented, but after accurate adjustment for age, this did not affect the risk estimates.

The lack of association with age at menarche is consistent with certain previous studies (Ji *et al*, 1996; Kreiger *et al*, 2001; Skinner *et al*, 2003; Navarro Silvera *et al*, 2005; Teras *et al*, 2005; Prizment *et al*, 2007), although others found positive (Duell and Holly, 2005; Lin *et al*, 2006) or inverse associations (Bueno de Mesquita *et al*, 1992; Kalapothaki *et al*, 1993; Fernandez *et al*, 1995; Hanley *et al*, 2001). A positive association with age at menopause was also found in a population-based case–control study (Duell and Holly, 2005). An inverse relation in a cohort study (Prizment *et al*, 2007) was heavily influenced by extreme values of age at natural menopause, while no association was found by others (Bueno de Mesquita *et al*, 1992; Ji *et al*, 1996; Kreiger *et al*, 2001; Skinner *et al*, 2003; Teras *et al*, 2005; Lin *et al*, 2006).

No significant relation with breastfeeding was found in a cohort study of younger women with shorter overall breastfeeding times (Skinner *et al*, 2003), while a case–control study, including extended breastfeeding periods, showed an inverse relation (Lo *et al*, 2007) consistent with our results.

A positive association with parity, seen only after adjustment for breastfeeding, implied that multiparous women who never breastfed carry an elevated risk. Multiparity and prolonged breastfeeding may be associated with a lower risk than in nulliparous women. Failure to take breastfeeding into account may partly explain previous disparate results. Skinner *et al* (2003), however, observed an inverse relation with parity even after adjustment for breastfeeding. A positive association with parity has been suggested as due to higher oestrogen levels, during pregnancy (Ji *et al*, 1996), and if such effects fade over time, they could produce an age interaction as seen in our study. As levels drop substantially during breastfeeding (Baird *et al*, 1979), a general risk-enhancing oestrogenic effect may also explain the inverse association with breastfeeding, in addition to the positive associations with abortions and age at menopause. Almost all women in our cohort were born too early to have used oral contraceptives or hormone replacement therapy, although these apparently do not affect risk (Teras *et al*, 2005). Alternatively, the breastfeeding effect may reflect the raised prolactin levels maintained at these times (Freeman *et al*, 2000) since prolactin causes a change in islet mass (Amaral *et al*, 2004). Although most tumours occur in the exocrine pancreas, the islets seem to play a role in carcinogenesis and may even harbour precursor cells (Hennig *et al*, 2004). Prolonged breastfeeding has recently been found to protect against subsequent type II diabetes (Stuebe *et al*, 2005) and diabetes may be a risk factor for pancreatic cancer (Inoue *et al*, 2006), but these relations do not readily explain our inverse association with breastfeeding.

It was not possible to adjust for cigarette smoking, a risk factor for pancreatic cancer. Among Norwegian women born in 1890–1894, the maximum proportion of smokers at any time was 10%, but this increased to about 40% among those born in 1925–1929 (Rønneberg *et al*, 1994). Most women (79%) lived in rural communities, with a considerably lower smoking prevalence (Zeiner-Henriksen, 1976), particularly in the oldest cohorts, containing the majority of cases. This was confirmed by lung cancer incidence in this cohort, rates being similar for women born before 1911 to non-smoking rates in Norway (Engeland *et al*, 1996) and for those born after 1910 were less than half the current rates (Cancer Registry of Norway, 2006).

Smoking is associated with earlier menopause by 1–2 years (Willett *et al*, 1983), so cannot explain our positive relation with age at menopause. The association with parity was most pronounced in the youngest birth cohorts, although risk estimates by age showed more variation than by birth cohorts (Table 6). This is unlikely to reflect confounding by smoking, which was first adopted in Norway by higher socioeconomic women (Kreyberg, 1954) who have fewer children than the average, whereas our breastfeeding effect was almost independent of birth cohort. Potential confounding with obesity can be excluded, as BMI was not associated with risk.

We found a higher risk among women reporting an abortion but no trend in risk with the number. Possibly those with several abortions reflected a tendency to miscarry early in pregnancy, whereas factors associated with full-term pregnancy also applied to the occasional later abortion. One early study (Lin and Kessler, 1981) showed an elevated risk in women after a spontaneous abortion, but no associations were found in studies (Bueno de Mesquita *et al*, 1992; Fernandez *et al*, 1995; Ji *et al*, 1996; Rosenblatt *et al*, 2006) that focused partly or wholly on induced abortions.

The lack of association with age at first birth is consistent with several studies (Skinner *et al*, 2003; Duell and Holly, 2005; Navarro Silvera *et al*, 2005; Teras *et al*, 2005; Lin *et al*, 2006; Prizment *et al*, 2007). However, two case–control studies (Fernandez *et al*, 1995; Kreiger *et al*, 2001) found positive associations and a large population-based study (Karlson *et al*, 1998) showed an inverse association, especially below 50 years, outside our age range.

The variety of findings in epidemiological studies of pancreatic cancer must raise the role of chance, although larger studies taking into account hormonal levels in different age groups might still be revealing.

## Figures and Tables

**Table 1:** Risk of pancreatic cancer by demographic factors

	**Person-years (× 10^5^)**	**Number of cases**	**RR (with 95% CI)**
*Age* (*years*)^a^			
50–59	4.09	36	0.42 (0.28–0.61)
60–69	5.14	112	1.00^b^
70–79	4.24	192	2.03 (1.60–2.58)
80–89	1.59	109	2.97 (2.25–3.93)
			
*Birth cohort* c			
1886–1900	2.19	104	1.00^b^
1901–1910	4.25	150	1.01 (0.79–1.30)
1911–1920	5.47	138	0.93 (0.72–1.22)
1921–1928	3.13	57	0.94 (0.66–1.33)
			
*County* d			
Nord-Trøndelag	5.14	164	1.00^b^
Aust-Agder	3.27	88	0.81 (0.63–1.06)
Vestfold	6.65	197	0.93 (0.76–1.15)

CI=confidence interval; RR=rate ratio.

a RRs adjusted for birth cohort.

b Reference category.

c RRs adjusted for age (in 2-year categories).

d RRs adjusted for age (in 5-year categories) and birth cohort.

**Table 2:** Risk of pancreatic cancer by menstrual history

	**Number of cases**	**RR (with 95% CI)**	***P*-value for linear trend**
*Age at menarche* (*years*)^a^			
⩽12	42	1.06 (0.75–1.50)	
13	82	1.15 (0.87–1.52)	
14	131	1.00^b^	
15	102	1.08 (0.83–1.40)	
⩾16	73	1.02 (0.77–1.36)	
Per year		0.99 (0.93–1.06)	0.86
			
*Age at menopause* (*years*)^a^			
⩽47	57	1.00^b^	
48–49	33	1.05 (0.68–1.63)	
50–51	58	1.47 (1.02–2.14)	
⩾52	44	1.37 (0.92–2.06)	
Per 2 years		1.08 (1.00–1.17)	0.03

CI=confidence interval; RR=rate ratio.

a RRs adjusted for age (in 5-year categories), birth cohort and county.

b Reference category.

**Table 3:** Risk of pancreatic cancer by parity and total duration of breastfeeding

		**Adjustment for age, birth cohort and county**		**Additional adjustment for parity or breastfeeding**	
	**Number of cases**	**RR (with 95% CI)**	***P*-value for linear trend**	**RR (with 95% CI)**	***P*-value for linear trend**
*Parity* a					
0	77	0.97 (0.70–1.35)		0.68 (0.45–1.04)	
1	67	1.00^b^		1.00^b^	
2	96	0.90 (0.66–1.23)		0.97 (0.69–1.35)	
3	93	1.25 (0.91–1.71)		1.47 (1.03–2.09)	
4	48	1.16 (0.80–1.69)		1.49 (0.98–2.26)	
⩾5	44	1.11 (0.75–1.64)		1.63 (1.01–2.64)	
Per delivery		1.03 (0.98–1.09)	0.21	1.13 (1.05–1.22)	0.002
					
*Duration of breastfeeding* (*months*)^c^					
0	40	1.00^b^		1.00^b^	
1–6	62	0.61 (0.41–0.91)		0.62 (0.42–0.93)	
7–12	73	0.66 (0.45–0.97)		0.66 (0.44–0.97)	
13–24	92	0.68 (0.47–0.98)		0.63 (0.42–0.93)	
25–36	50	0.65 (0.43–0.99)		0.50 (0.31–0.78)	
>36	31	0.56 (0.34–0.90)		0.40 (0.23–0.70)	
Per 12 months		0.95 (0.87–1.03)	0.23	0.87 (0.78–0.97)	0.01

CI=confidence interval; RR=rate ratio.

a Among all women with known parity and duration of breastfeeding.

b Reference category.

c Among parous women with known duration of breastfeeding.

**Table 4:** Risk of pancreatic cancer by age at deliveries^a^

	**Number of cases**	**RR (with 95% CI)**	***P*-value for linear trend**
*Age at delivery* (*years*), *uniparous women*			
⩽24	13	0.92 (0.46–1.84)	
25–29	22	1.00^b^	
30–34	13	0.67 (0.33–1.33)	
⩾35	15	0.94 (0.47–1.87)	
Per 5 years		0.96 (0.77–1.19)	0.71
			
*Age at first delivery* (*years*), *multiparous women*^c^			
⩽24	121	1.11 (0.82–1.49)	
25–29	98	1.00^b^	
30–34	38	0.98 (0.66–1.46)	
⩾35	14	1.26 (0.67–2.34)	
Per 5 years		1.01 (0.84–1.21)	0.93
			
*Age at last delivery* (*years*), *multiparous women*^d^			
⩽29	53	0.86 (0.60–1.24)	
30–34	94	1.00^b^	
35–39	82	0.89 (0.64–1.23)	
⩾40	42	0.86 (0.56–1.33)	
Per 5 years		1.05 (0.89–1.24)	0.54

CI=confidence interval; RR=rate ratio.

a Among parous women with known age at deliveries and duration of breastfeeding; rate ratios adjusted for age (in 5-year categories), birth cohort, county and duration of breastfeeding.

b Reference category.

c Additional adjustment for parity and age at last delivery.

d Additional adjustment for parity and age at first delivery.

**Table 5:** Risk of pancreatic cancer by number of abortions^a^

	**Number of cases**	**RR (with 95% CI)**	***P*-value**
*Number of abortions*			
0	304	1.00^b^	
1	81	1.42 (1.10–1.82)	
2	21	1.27 (0.81–1.98)	
⩾3	6	0.89 (0.39–1.99)	
Per abortion		1.10 (0.96–1.25)	0.18^c^
Any abortion *vs* no abortion		1.34 (1.07–1.68)	0.01

CI=confidence interval; RR=rate ratio.

a RRs adjusted for age (in 5-year categories), birth cohort, county, parity and duration of breastfeeding.

b Reference category.

c *P*-value for linear trend.

**Table 6:** Risks by parity, duration of breastfeeding and age at menopause in categories of age and birth cohort

	**Parity^a^**		**Duration of breastfeeding^b^**		**Age at menopause^c^**	
	**RR per delivery (with 95% CI)**	***P*-value for linear interaction^d^**	**RR per 12 months (with 95% CI)**	***P*-value for linear interaction^d^**	**RR per 2 years (with 95% CI)**	***P*-value for linear interaction^d^**
*Age (years)*		0.08		0.79		0.15
50–69	1.23 (1.09–1.40)		0.92 (0.75–1.11)		1.14 (0.96–1.34)	
70–79	1.12 (1.00–1.25)		0.79 (0.67–0.93)		1.12 (1.00–1.26)	
80–89	1.03 (0.86–1.23)		0.98 (0.78–1.22)		1.01 (0.90–1.14)	
						
*Birth cohort*		0.36		0.63		0.83
1886–1900	1.09 (0.95–1.26)		0.87 (0.72–1.05)		1.07 (0.97–1.18)	
1901–1910	1.03 (0.89–1.20)		0.89 (0.73–1.09)		1.12 (0.99–1.25)	
1911–1928	1.26 (1.12–1.41)		0.85 (0.71–1.02)		—e	

CI=confidence interval; RR=rate ratio.

a Among all women with known parity and duration of breastfeeding; adjustment for age (in 5-year categories), birth cohort, county and breastfeeding.

b Among parous women with known duration of breastfeeding; adjustment for age, birth cohort, county and parity.

c Among women with known age at menopause; adjustment for age, birth cohort and county.

d Between original values of risk factor and 5-year scores for age or 10-year scores for birth cohort.

e No cases with known age at menopause in this category.
